# A Growing Armamentarium for the Robotic Pancreas Surgeon?

**DOI:** 10.1097/AS9.0000000000000282

**Published:** 2023-05-05

**Authors:** Gregory C. Wilson

**Affiliations:** From the Department of surgery, Division of Surgical Oncology, University of Cincinnati Medical Center, Cincinnati

In this current issue, an article by Dr. Fong et al.^1^ describes the technical details for the routine use of pancreatoscopy during robotic pancreas surgery for main duct assessment in intraductal papillary mucinous neoplasms. Robotic-assisted surgery has emerged as a revolutionary technology that offers new possibilities for performing complex surgeries, including pancreaticoduodenectomy and other pancreatic resections.^2^ The routine use of robotics in pancreatic surgery has steadily increased over the past decade and has been widely disseminated throughout the world.^3^ The technology and toolset associated with robotic surgery have many advantages over previous minimally-invasive approaches however, one clear deficit of robotic-assisted surgery is the lack of haptic feedback. This limitation is overcome by rigorous surgeon training, improved visualization, and high-quality patient imaging. Preoperative cross-sectional imaging is standard for all pancreatic resections but high-quality intraoperative assessments specifically in robotic surgery with the use of a drop-in ultrasound probe can be just as critical. The authors here provide another method for intraoperative visualization with the novel use of pancreatoscopy to visualize the lumen of the pancreatic duct during surgery.

Patients undergoing surgery for intraductal papillary mucinous neoplasm can have margin positive resections in up to 20% of resections.^4,5^ Although we know that separate lesions arising within the “field defect” are more problematic than low-grade margin positivity postoperatively,^6^ a margin negative resection remains the primary objective of surgery. The use of intraoperative pancreatoscopy during robotic pancreas resection for margin assessment is an attractive use of this technology. It allows for a rapid and direct assessment of the pancreatic duct and determination of a clean transection plane when we would otherwise rely on preoperative imaging, anatomic landmarks, and occasionally intraoperative ultrasound. Additionally, there is another more common clinical scenario to consider, which is the extent of ductal dilation present beyond the neck in a proximal lesion or extension into the neck/head for left-sided lesions that with conventional imaging would portend a more extended resection. The robotic platform and intraoperative pancreatoscopy could be incorporated in this setting to determine if a standard resection is feasible and appropriate. Any additional parenchyma spared could have an immediate quality of life impact on the patient.

A word of caution should be exercised whenever the implementation of new technology is considered. Although the reported benefits of pancreatoscopy are outlined within the article, one must also consider the converse. Specifically, in the setting of a false positive finding on pancreatoscopy leading to a more extensive resection and potentially adding to the short- and long-term morbidity associated with the surgery. The metrics of this test, including sensitivity/specificity, number needed to treat, etc, should be understood before widespread adoption ensues. Additionally, the cost and access to the technology must be considered. The proposed method requires the use of a single-use disposable scope as well as the expertise of an advanced endoscopist to interpret the images of the duct. Both of these requirements result in a high cost of entry for routine intraoperative pancreatoscopy and may be prohibitive in many settings. Fortunately, some of these questions/concerns are already being addressed with the ongoing proposed trial cited by the authors and they should be applauded for seeking these answers. Like any new technological advancement, the key here will be understanding the right time and place for intraoperative pancreatoscopy.
